# Amyloid precursor protein drives down-regulation of mitochondrial oxidative phosphorylation independent of amyloid beta

**DOI:** 10.1038/s41598-017-10233-0

**Published:** 2017-08-29

**Authors:** M. Isabel G. Lopez Sanchez, Hayley S. Waugh, Andrew Tsatsanis, Bruce X. Wong, Jonathan G. Crowston, James A. Duce, Ian A. Trounce

**Affiliations:** 1 Centre for Eye Research Australia, 75 Commercial Road, Melbourne, 3004 Victoria Australia; 2 Department of Surgery, Ophthalmology, University of Melbourne, Victoria, Australia; 3 School of Biomedical Sciences, Faculty of Biological Sciences, University of Leeds, Leeds, West Yorkshire LS2 9JT United Kingdom; 4 Oxidation Biology Unit, The Florey Institute of Neuroscience and Mental Health, University of Melbourne, 30 Royal Parade, Parkville, 3052 Victoria Australia

## Abstract

Amyloid precursor protein (APP) and its extracellular domain, soluble APP alpha (sAPPα) play important physiological and neuroprotective roles. However, rare forms of familial Alzheimer’s disease are associated with mutations in *APP* that increase toxic amyloidogenic cleavage of APP and produce amyloid beta (Aβ) at the expense of sAPPα and other non-amyloidogenic fragments. Although mitochondrial dysfunction has become an established hallmark of neurotoxicity, the link between Aβ and mitochondrial function is unclear. In this study we investigated the effects of increased levels of neuronal APP or Aβ on mitochondrial metabolism and gene expression, in human SH-SY5Y neuroblastoma cells. Increased non-amyloidogenic processing of APP, but not Aβ, profoundly decreased respiration and enhanced glycolysis, while mitochondrial DNA (mtDNA) transcripts were decreased, without detrimental effects to cell growth. These effects cannot be ascribed to Aβ toxicity, since higher levels of endogenous Aβ in our models do not cause oxidative phosphorylation (OXPHOS) perturbations. Similarly, chemical inhibition of β-secretase decreased mitochondrial respiration, suggesting that non-amyloidogenic processing of APP may be responsible for mitochondrial changes. Our results have two important implications, the need for caution in the interpretation of mitochondrial perturbations in models where APP is overexpressed, and a potential role of sAPPα or other non-amyloid APP fragments as acute modulators of mitochondrial metabolism.

## Introduction

Despite reports of a protective role in neuronal damage^1–5^, research into the cellular function of APP has been highly influenced by an Aβ-centric focus. APP is processed from its membrane-bound holoform via two pathways, the predominant non-amyloidogenic processing or the much less common amyloidogenic pathway, which generates Aβ. The non-amyloidogenic pathway involves cleavage of APP by α-secretase, producing a large N-terminal ectodomain, sAPPα, which is secreted into the extracellular medium^6^, and an 83 amino-acid C-terminal fragment, which is subsequently cleaved by γ-secretase, producing a short peptide called p3^7^. Cleavage by α-secretase occurs at a position within the sequence of Aβ, therefore precluding its formation^8^. *In vitro* and *in vivo* work has demonstrated that sAPPα is neuroprotective and neurotrophic^1, 2, 9–13^ and has been shown to protect against Aβ toxicity^14–16^. In contrast, the amyloidogenic pathway involves cleavage by β-secretase, resulting in the release of soluble APP β (sAPPβ) into the extracellular medium, and a 99 amino acid C-terminal fragment in the membrane^17^. Subsequent cleavage of this fragment by γ-secretase generates the Aβ peptide. Most of the Aβ produced is 40 amino acid residues in length (Aβ_40_) but a small proportion (~10%) is a 42-residue variant (Aβ_42_) and the predominant species found in cerebral plaques in Alzheimer’s disease^18, 19^.

Rare, autosomal dominant forms of familial Alzheimer’s disease are caused by mutations in *APP*. The double mutation (K670N/M671L) in APP, which increases β-secretase affinity and thus enhances Aβ production, is associated with familial Alzheimer’s disease in Swedish pedigrees^20^. Since its identification, the Swedish mutation has been introduced to numerous models of amyloid pathology, and a link between Aβ and mitochondrial dysfunction has been reported, including effects on OXPHOS and ATP production^21–24^, Although mitochondrial dysfunction has become an established hallmark of neuronal toxicity, the link between Aβ and mitochondrial function is still unclear^25^.

In this study we compared the effects of increased levels of neuronal APP wild-type or Swedish mutant APP expression on mitochondrial OXPHOS metabolism, by examining changes in mitochondrial respiration, protein expression, enzymatic activity and gene expression in the SH-SY5Y human neuroblastoma cell line.

## Results

### Increased levels of APP wild-type decrease mitochondrial respiration and enhance glycolysis

**Figure 1:**
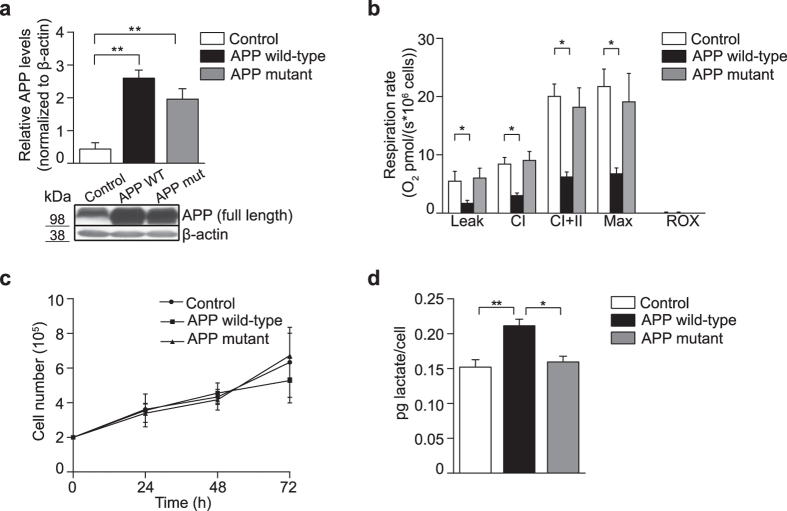
Increased APP wild-type levels decrease mitochondrial respiration and enhance glycolysis. (**a**) Densitometric measurements and representative immunoblot show that total APP protein levels are significantly higher in human neuroblastoma SH-SY5Y cells stably-transfected with APP wild-type (APP WT) or APP mutant (APP mut) relative to the empty vector control. β-actin was used as a loading control. (**b**) Mitochondrial respiration is decreased in digitonin-permeabilized cells that express APP wild-type compared to control. Leak, CI = complex I-driven, CI + II = convergent complex I + II, Max = maximal uncoupled respiration, ROX = residual oxygen consumption. (**c**) Growth curves obtained using the trypan blue exclusion assay show no detectable changes in cell proliferation between control, APP wild-type and APP mutant cells over 72 h. (**d**) Lactate production is significantly increased in cells that overexpress APP wild-type, relative to both control and APP mutant cells after 48 h in culture. Data is presented as mean ± SD (n = 3); *P < 0.05 and **P < 0.01 by one-way ANOVA.

To investigate the effects of APP or Aβ on mitochondrial metabolism, human neuroblastoma SH-SY5Y cells were transfected with an empty vector (control) or with the neuronal-specific APP wild-type isoform (APP wild-type) or Swedish APP mutant (APP mutant) cDNA, which increases Aβ production. Transfection efficiency was assessed by immunoblotting, to show that APP wild-type and APP mutant cells both express significantly higher levels of APP compared to control (Fig. 1a; **P < 0.01).

Mitochondrial OXPHOS metabolism was first examined by measuring oxygen consumption in a high-resolution Oroboros oxygraph-2k (Fig. 1b). In digitonin-permeabilized cells, we measured the leak respiratory rate (leak), complex I-driven (CI) and convergent complex I + II-linked (CI + II) rates by providing specific substrates for these complexes in the presence of excess ADP, followed by addition of the uncoupler CCCP to detect maximal uncoupled respiration (Max). Residual oxygen consumption, also known as non-mitochondrial respiration, was confirmed to be negligible by addition of rotenone and antimycin A. We observed a significant decrease of overall respiration in cells overexpressing APP wild-type compared with control (Fig. 1b; *P < 0.05), as previously reported^26^. However, no change was detected in cells that overexpress APP mutant relative to control (Fig. 1b). Cell proliferation was measured in control, APP wild-type and APP mutant cells over 72 h using the trypan blue exclusion assay (Fig. 1c), and confirmed that overexpression of either form of APP does not lead to decreased proliferation or cell death within these experimental parameters.

Mammalian cells rely on both aerobic respiration and glycolysis to produce ATP and sustain cellular function and proliferation. As we observed a decrease in mitochondrial respiration in cells overexpressing APP wild-type relative to control, we measured lactate production as a surrogate marker of glycolytic metabolism (Fig. 1d). Lactate production was significantly increased in APP wild-type cells compared with control and APP mutant cells after 48 h (Fig. 1d; *P < 0.05 and **P < 0.01). These results indicate that cells expressing higher levels of APP wild-type undergo a metabolic shift to glycolysis in response to the down-regulation of respiration that is not detrimental to cellular proliferation, whereas higher expression of APP mutant does not induce changes in respiration, glycolysis or cell proliferation.

### OXPHOS protein levels and complex IV enzymatic activity are decreased in APP wild-type cells

**Figure 2:**
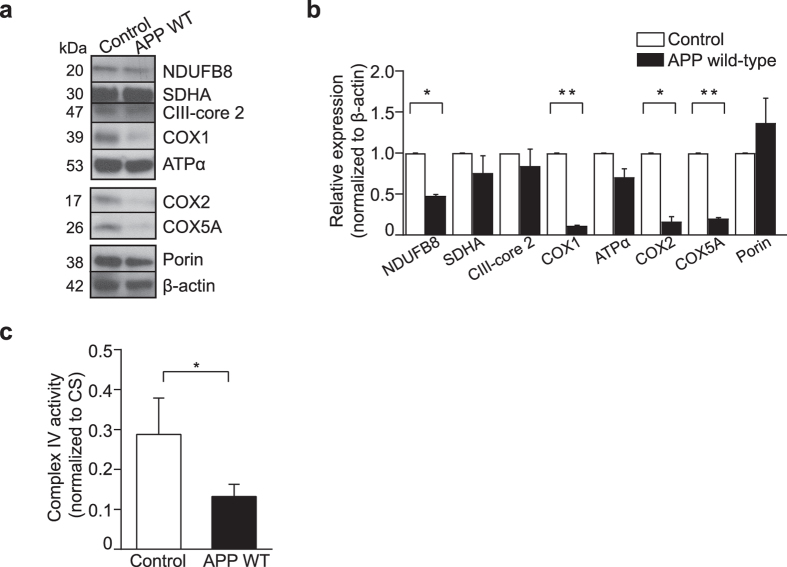
OXPHOS complex IV protein levels and enzymatic activity are decreased in APP wild-type cells. (**a)** Representative immunoblot of OXPHOS protein levels in lysates from cells expressing APP wild-type (APP WT) compared to control. Porin was used as a marker for mitochondrial content and β-actin was used as a loading control. (**b)** Densitometric analysis of immunoblot images showing a significant decrease in the expression of NDUFB8, COX1, COX2 and COX5 A in APP wild-type cell lysates compared to control. (**c)** OXPHOS complex IV enzymatic activity is significantly decreased in mitochondria from cells overexpressing APP wild-type relative to control. Complex IV specific activity was normalized to protein content and to citrate synthase (CS) activity. All data is presented as mean ± SD (n = 3); *P < 0.05 and **P < 0.01 by paired, two-tailed Student’s *t*-test.

We assessed whether decreased respiration in cells expressing APP wild-type reflected changes in OXPHOS protein levels, by quantifying steady-state levels of key proteins of each OXPHOS complex. Nuclear DNA-encoded NADH dehydrogenase 1 β sub-complex subunit 8 (NDUFB8; complex I), succinate dehydrogenase flavoprotein subunit (SDHA; complex II), complex III core protein 2 (CIII-core 2; complex III), ATP synthase subunit α (ATPα; ATP synthase, complex V), cytochrome *c* oxidase subunit 5 A (COX5 A; complex IV) and mtDNA-encoded cytochrome *c* oxidase subunits 1 and 2 (COX1 and COX2; complex IV) were measured in whole cell lysates by immunoblotting, using porin as a marker for mitochondrial content and β-actin as a loading control (Fig. 2a and b). NDUFB8, COX1, COX2 and COX5 A were significantly decreased in APP wild-type cells compared to control, whereas the levels of the other OXPHOS subunits tested remained unchanged relative to control (Fig. 2b; *P < 0.05 and **P < 0.01). This was further supported by measurements of OXPHOS protein levels by immunoblotting in mitochondrial preparations (Supplementary Fig. 1), which showed significant decreases in COX1, COX2 and COX5A in mitochondria from APP wild-type cells relative to control.

Given the significant reduction in complex IV protein levels in cells overexpressing APP wild-type, we examined complex IV enzymatic activity in isolated mitochondria. Complex IV activity measurements were normalized to citrate synthase (CS) activity to account for changes in mitochondrial density, and similar to a previous report^26^, found to be significantly decreased in mitochondria from cells that express APP wild-type relative to control (Fig. 2c; *P < 0.05). Together, the reduction in steady-state protein levels and enzymatic activity indicates that decreased mitochondrial respiration in APP wild-type cells is linked to a down-regulation of OXPHOS complexes I and IV. However, in these experimental parameters this is not detrimental to cellular health.

### Expression of APP wild-type decreases mitochondrial transcripts and increases mtDNA copy number

**Figure 3:**
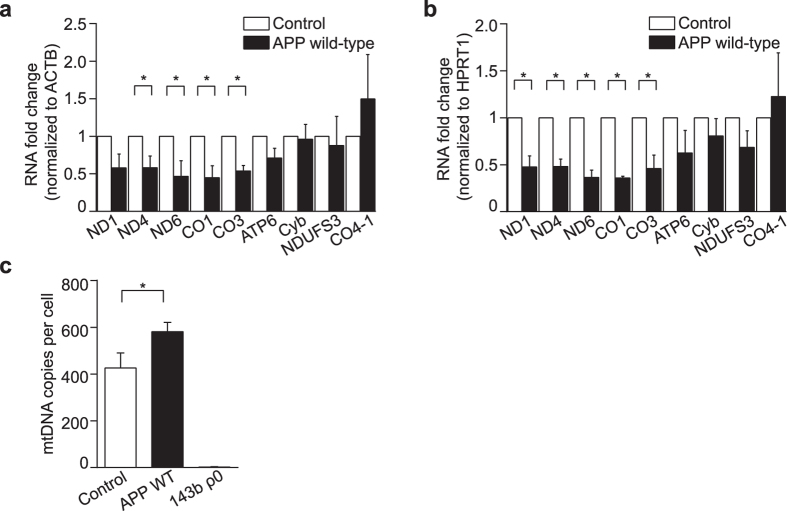
Expression of APP wild-type decreases mitochondrial transcripts and increases mtDNA copy number. (**a)** Mitochondrial OXPHOS transcripts were quantified by qRT-PCR. MtDNA-encoded ND1, ND4, ND6 (complex I), CO1, CO3 (complex IV), ATP6 (ATP synthase/complex V), Cyb (complex III) and nuclear DNA-encoded NDUFS3 (complex I) or CO4-1 (complex IV) mRNA levels were normalized to ACTB or HPRT1 (**b**). A significant decrease was detected in mtDNA-encoded ND4, ND6, CO1 and CO3 mRNAs in APP wild-type (APP WT) cells relative to control. ND1 transcript was significantly decreased when using HPRT1 as a reference only. (**c)** MtDNA copy number is significantly increased in APP wild-type cells relative to control. Human osteosarcoma 143B ρ0 cells devoid of mtDNA were used as a negative control. Data is presented as mean ± SD (n = 3); *P < 0.05 by paired, two-tailed Student’s *t*-test.

We investigated whether the OXPHOS changes in APP wild-type overexpressing cells reflected an underlying regulation of mitochondrial gene expression, by measuring changes in mitochondrial transcript abundance, and mtDNA copy number. OXPHOS transcripts were measured by qRT-PCR on mtDNA-encoded NADH dehydrogenase subunits 1, 4 and 6 (ND1, ND4 and ND6; complex I), cytochrome *c* oxidase subunits 1 and 3 (CO1 and CO3, complex IV), ATP synthase 6 (ATP6; ATP synthase/complex V), cytochrome *b* (Cyb; complex III), as well as nuclear DNA-encoded NADH:ubiquinone oxidoreductase core subunit S3 (NDUFS3; complex I) and cytochrome *c* oxidase subunit COX IV-1 (CO4-1; complex IV). Quantification was normalized to β-actin (ACTB; Fig. 3a) or hypoxanthine phosphoribosyl transferase 1 (HPRT1, Fig. 3b), to assure that changes measured were independent of the reference gene selected^27^. Transcript analysis showed a significant decrease in ND4, ND6, CO1 and CO3 mRNAs in APP wild-type cells relative to control (Fig. 3a and b; *P < 0.05). ND1 transcript was significantly decreased when using HPRT1 as a reference only, whereas no changes were detected in nuclear DNA-encoded NDUFS3 and CO4-1, or mtDNA-encoded ATP6 and Cyb mRNAs.

MtDNA copy number per cell was quantified by qPCR using nuclear *ACTB* as a marker of diploid genome content and *MT-ND2* as a marker for the mitochondrial genome (Fig. 3c). Human osteosarcoma 143B ρ0 cells, which are devoid of mtDNA^28^, were used as a negative control. Unexpectedly, we observed a significant increase in mtDNA copy number in APP wild-type cells relative to control (Fig. 3c; *P < 0.05), despite the observed decrease in steady-state levels of mtDNA-encoded transcripts. This suggests that APP wild-type cells activate mtDNA replication, despite mitochondrial transcripts being down-regulated, possibly as a result of post-transcriptional regulation processes, which have been shown to modulate the expression of specific mitochondrial transcripts^29, 30^.

These results indicate a down-regulation of mitochondrial OXPHOS metabolism in cells that express APP wild-type, accompanied by a metabolic switch that enhances glycolytic metabolism in response to diminished respiration, without detriment to cellular proliferation. Decreases in overall respiration are likely driven by a reduction of complex I and IV protein levels consequent to decreased mtDNA transcription in cells overexpressing APP wild-type.

### Down-regulation of mitochondrial OXPHOS is independent of Aβ levels

**Figure 4:**
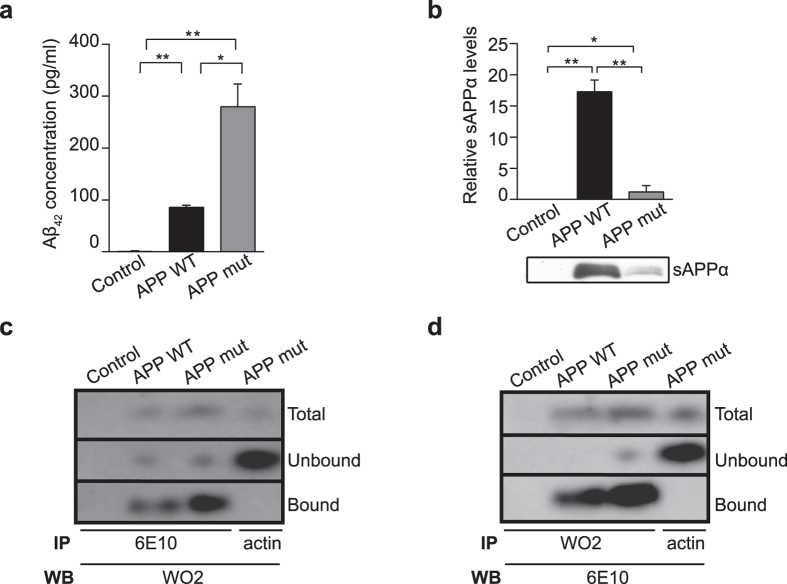
APP metabolite levels in cells expressing APP wild-type or APP mutant. (**a)** Aβ_42_ levels were significantly increased in cells expressing APP wild-type (APP WT) and APP mutant (APP mut) compared with control, and significantly higher in APP mutant compared to APP wild-type cells. Aβ_42_ levels were measured by ELISA in medium collected from control, APP wild-type or APP mutant cells. (**b)** Densitometry analysis and representative immunoblot show a significant increase in the expression of sAPPα in medium collected from cells expressing APP wild-type and APP mutant compared with control. However, significantly higher sAPPα levels were detected in APP wild-type cells compared to APP mutant cells. Results indicate average of duplicate wells of each sample, from three independent medium collections per cell line, seeded at equal densities. Data is presented as mean ± SD (n = 3); *P < 0.05 and **P < 0.01 by one-way ANOVA. (**c)** From homogenates of control, APP wild-type (APP WT) or APP mutant (APP mut) cells, proteins were fractionated by interaction with antibodies to detect Aβ (6E10 or WO2, (**d**). “Total” represents 15 μg of the 250 μg total protein used for immunoprecipitation, “bound” fraction or other “unbound” proteins. In immunoprecipitated samples, visualization was carried out by immunoblot with an alternative antibody to Aβ (WO2 or 6E10). Aβ levels were undetectable in control cells, whereas APP mutant cells had elevated Aβ compared to wild-type. An antibody to β-actin was used to confirm antibody selectivity for Aβ.

Decreases in OXPHOS complex IV activity in SH-SY5Y cells expressing APP wild-type have previously been attributed to increased Aβ levels^26, 31^. We measured Aβ_42_ levels by ELISA in medium collected from cells expressing endogenous APP or overexpressing APP wild-type or APP mutant (Fig. 4a). As expected, cells overexpressing APP wild-type or APP mutant both secrete more Aβ_42_ compared to control cells (Fig. 4a; *P < 0.05 and **P < 0.01), but consistent with previous reports on increased amyloidogenic cleavage in cells that express APP mutant^32–35^, Aβ_42_ levels were significantly increased compared to APP wild-type cells. Importantly, despite an increased Aβ load, this does not affect OXPHOS in APP mutant cells, as shown in functional assays carried out on the same cells (Fig. 1b). The alternative non-amyloidogenic cleavage of full-length APP that releases sAPPα was also measured by immunoblotting and showed a significantly higher sAPPα production in medium from cells overexpressing APP wild-type compared with APP mutant and control cells - that express endogenous levels of APP wild-type (Fig. 4b; *P < 0.05 and **P < 0.01). We also visualized cellular Aβ using an immunoprecipitation approach. As predicted, higher Aβ levels were observed in cells overexpressing the APP mutant, compared to APP wild-type and control (Fig. 4c and d). This indicates that while stably-transfected cell lines both secrete higher levels of Aβ and sAPPα relative to control cells, the ratios of these proteins are changed by the introduction of the mutation into APP. Thereby cells expressing APP mutant produce higher levels of Aβ, whereas APP wild-type overexpressing cells secrete higher levels of sAPPα. As mitochondrial OXPHOS is decreased only in cells overexpressing APP wild-type, our data suggest that these functional changes are independent of increased Aβ levels.

### Inhibition of β-secretase increases sAPPα and decreases mitochondrial respiration in cells expressing APP mutant

**Figure 5:**
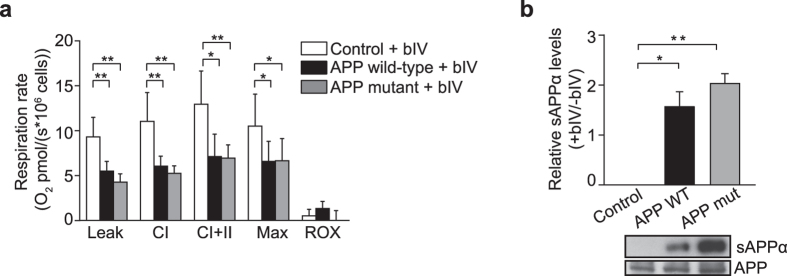
Inhibition of β-secretase decreases mitochondrial respiration in APP mutant cells. (**a)** Mitochondrial respiration was decreased in digitonin-permeabilized APP wild-type (APP WT) and APP mutant (APP mut) cells when incubated with 1 μM β-secretase inhibitor IV (bIV) for 24 h relative to control. Leak, CI = complex I-driven, CI + II = convergent complex I + II rates, Max = maximal uncoupled respiration, ROX = residual oxygen consumption. (**b)** Densitometry analysis and representative immunoblot show a significant increase in the expression of sAPPα in medium collected from cells expressing APP wild-type or APP mutant following incubation with 1 μM β-secretase inhibitor IV for 24 h relative to untreated cells. Data is presented as mean ± SD (n = 3); *P < 0.05 and **P < 0.01 by one-way ANOVA.

To confirm that decreased respiration in APP wild-type cells is independent of Aβ, amyloidogenic processing of APP wild-type and APP mutant cells was disrupted by chemical inhibition of the β-secretase activity required for amyloidogenic cleavage of APP. Upon β-secretase inhibition (bIV; 1 µM), significant decreases in respiratory rates were evident in both APP wild-type and APP mutant overexpressing cells, relative to control (Fig. 5a; *P < 0.05 and **P < 0.01). Compared to untreated cells (Fig. 1b), the addition of bIV resulted in respiration capacity decreasing in the APP mutant cells to match the APP wild-type cells (Fig. 5a). Some decrease in respiration was observed in control cells treated with bIV (Supplementary Table 3), that may indicate non-specific effects on respiration, although this decrease did not mask the clear shift to lower respiration in the APP mutant cells treated with bIV. Enhanced non-amyloidogenic processing upon treatment with bIV in APP mutant cells was confirmed by increased levels of sAPPα in media collected from treated cells, relative to cells without bIV treatment (Fig. 5b; *P < 0.05 and **P < 0.01). The significant decrease in mitochondrial respiration in cells that express APP wild-type only, or APP wild-type and APP mutant cells treated with bIV, support the notion that these functional changes are independent of increased Aβ levels, and are instead associated with the non-amyloidogenic processing of APP.

## Discussion

Our results indicate that increased APP wild-type levels lead to a down-regulation of OXPHOS, and enhanced glycolytic metabolism. This metabolic switch may enable cells expressing higher APP wild-type levels to sustain proliferation at similar levels to control cells and cells overexpressing APP mutant. Furthermore, we identified changes in mitochondrial gene expression in cells that express APP wild-type, with decreased levels of most mtDNA-encoded transcripts studied. We demonstrated that the effects seen in OXPHOS are independent of Aβ, as cells transfected with APP mutant exhibit higher Aβ levels than cells expressing APP wild-type or control, without altering OXPHOS function. Finally, we showed that chemical inhibition of β-secretase increases sAPPα levels in APP mutant cells, and results in a significant decrease in respiration that is similar to APP wild-type cells with or without β-secretase inhibition.

Complex IV enzymatic activity is decreased in platelet and brain mitochondria from patients with sporadic Alzheimer’s disease^36–38^, although the specificity of the decrease has been questioned^39^. This finding prompted efforts to explore whether decreased complex IV could be directly linked to effects of Aβ, with positive findings *in vitro* ^40, 41^. Because Aβ levels are greatly increased in mouse and *in vitro* models of amyloid pathology^26, 42, 43^, it has become generally accepted that Aβ is responsible for mitochondrial impairments, especially of complex IV. Unlike sporadic Alzheimer’s disease, these experimental models express varying levels of APP, either in its wild-type form or as Mendelian variants associated with familial Alzheimer’s disease. The levels of sAPPα have not been measured systematically in these studies, and we propose that greatly increased levels of sAPPα will occur even despite increased amyloidogenic cleavage.

To date all APP mutations linked to amyloid pathology alter the proteolytic processing of the protein^44^, and result in an increased production of Aβ, at the expense of non-amyloidogenic APP metabolites, such as sAPPα. Our data highlight the need for a better understanding of the cellular function of APP and its metabolites, to elucidate their potential contribution to mitochondrial homeostasis, especially in regards to the mechanism by which complex IV is down-regulated. Further work is needed to determine whether intracellular sAPPα, or other non-amyloidogenic APP fragments such as carboxy terminal fragment 83 or P3, or full-length APP^45^, or a receptor-mediated sAPPα effect is responsible for the changes seen on OXPHOS. Our results contradict the hypothesis that OXPHOS down-regulation or “impairment” is a cellular response to the toxic effects of Aβ, as suggested by others^24, 26, 43, 46–48^.

We speculate that APP-mediated down-regulation of OXPHOS may be a protective mechanism to limit OXPHOS-derived reactive oxygen species (ROS) in susceptible neurons. OXPHOS is the main cellular source of ROS^49^, which under normal physiological conditions, plays an important role in retrograde signaling from the organelle to the cytosol and nucleus in a tightly regulated process^49, 50^. However, in disease, increased ROS production has been linked to chronic oxidative damage and shown to induce cell death and neurotoxicity^51, 52^. Further work will elucidate whether APP-induced down-regulation of OXPHOS is a protective mechanism in the context of neuronal injury, where up-regulation and translocation of APP to injury sites is established^2, 53–56^.

## Methods

### Cell culture and plasmid transfection

Human SH-SY5Y neuroblastoma cells were cultured in RPMI 1640 medium (Thermo Fisher Scientific, Cat. #11875) and supplemented with 10% fetal bovine serum (FBS), 100 U/ml penicillin and 100 μg/ml streptomycin sulfate (basal medium) in a humidified incubator at 37 °C and 5% CO_2_. SH-SY5Y cells were stably transfected with a pIRESpuro2 empty vector control (Clontech Laboratories Inc., Takara-Bio Inc., Japan; control) or the vector containing either the full-length APP_695_ isoform as a wild-type (APP wild-type) or harboring the Swedish double mutant (K670N/M671L; APP mutant) using Lipofectamine 2000 (Thermo Fisher Scientific) according to manufacturer’s instructions. Plasmid expression was maintained by growing cells in RPMI 1640 selective medium containing 2 μg/ml puromycin (Sigma-Aldrich) and confirmed by immunoblotting.

### Chemical inhibition of β-secretase activity

Cells were incubated with 1 μM β-secretase inhibitor IV (bIV; Merck Millipore) for 24 h and efficiency of β-secretase activity inhibition was confirmed by immunoblotting, through detection of APP fragments in cell lysates or medium collected from treated cells.

### Mitochondrial enrichment

Mitochondria were extracted from freshly harvested cells as described previously^57^. Protein concentration was determined by the bicinchoninic acid assay (BCA) using a BCA Protein Assay kit (Thermo Fisher Scientific, Cat. #23225).

### ELISA of extracellular Aβ_42_

Secreted Aβ_42_ was measured in cell culture medium samples using a human Aβ_42_ ELISA kit (Thermo Fisher Scientific, Cat. #KHB3441) in cells plated at an equal density. Raw absorbance data was normalized to blank wells. A standard curve was generated by linear regression analysis and used to calculate the amount of Aβ_42_ in each sample. Assay results indicate averages of duplicate wells of each sample and three independent collections of medium from each cell line.

### Immunoprecipitation of cellular Aβ

SH-SY5Y cells stably transfected with empty vector, APP wild-type or APP mutant were lysed in RIPA buffer containing protease inhibitor cocktail (Thermo Fisher Scientific). Lysates were clarified (14,000 × *g*, 20 min) and supernatant protein concentration assayed. Immunoprecipitation of Aβ was performed using Dynabeads Protein-G Immunoprecipitation kit (Life Technologies) with modification to the manufacturer’s protocol. For Aβ capture an antibody raised to epitopes in the Aβ domain of APP (1:200, mouse anti-W02; or 1 μg, mouse anti-6E10; BioLegend) was pre-incubated with protein G beads for 2 h at room temperature. Beads complexed with the capture antibody were then washed 3 times for 5 min in wash buffer (TBS-0.1% Tween, pH 8.0) before the sample (250 μg) was added and incubated with continual movement (24 h, 4 °C on a rotary wheel). Unbound proteins from the supernatant were collected and beads were washed 5 times in wash buffer. Proteins still bound to beads were eluted with SDS-PAGE loading buffer (10 min, 70 °C). The bound and unbound proteins were separated by 4–12% SDS-PAGE (Bis-Tris, Invitrogen) and visualized by immunoblotting using an Aβ detection antibody that differed from the one used for immunoprecipitation capture.

### Immunoblotting

Immunoblotting was performed from culture medium, total cellular lysate, mitochondrial preparations and immunoprecipitates from transfected cells. Sample preparation and SDS-PAGE information are detailed as Supplementary Information. Proteins of interest were detected using the antibodies indicated in Supplementary Table 1 and visualized by enhanced chemiluminescence detection on film (Amersham GE Healthcare, Cat. #RPN2106). Protein expression was quantified by relative densitometry using ImageJ software (imagej.nih.gov).

### Cell proliferation

Cell proliferation was measured by the trypan blue exclusion assay. Equal numbers of cells (2 × 10^5^) were seeded in plates for 24, 48 and 72 h. At these time points cells were detached, washed in PBS and suspended in trypan blue solution (Thermo Fisher Scientific, Cat. #1525006) at a 1:1 ratio and counted using a hemocytometer.

### Lactate production

Lactate was measured in cell culture medium using a commercial kit (Sigma-Aldrich, Cat. #MAK065). Cells seeded in triplicate (1 × 10^6^) were incubated overnight in basal medium before replacement with phenol red-free RPMI containing 5% FBS, 22.5 mM glucose, 0.2 mM uridine and 1 mM sodium pyruvate. Phenol red-free medium was used to prevent interference with colorimetric measurements and serum in the medium was reduced to 5% to minimize interference from serum-based lactate dehydrogenase activity. After 48 h, 10 μl of medium was collected to measure lactate, using an absorbance plate reader at 450 nm (ELx800, Biotek) and compared with a lactate standard curve. Measurements were corrected for assay background and normalized to cell number after incubation in each sample.

### High-resolution mitochondrial respiration analysis

Leak, ADP-stimulated complex I-driven, convergent complex I + II, maximal uncoupled and residual oxygen (ROX) consumption rates were measured in stably-transfected cells. Respiration was measured by sequential injection of glutamate (10 mM), malate (2 mM), digitonin (10 µg/ml; leak respiration), ADP (1 mM; ADP-stimulated complex I), succinate (10 mM; complex I + II), CCCP (1.5 µM; maximal uncoupled), rotenone (5 µM) and antimycin A (2 µM; ROX), in a high-resolution Oroboros Oxygraph 2 K (Oroboros Instruments) as described previously^58^. Measurements were normalized to cell number per chamber and data was analyzed using the Datlab2 software (Oroboros Instruments).

### Complex IV enzymatic activity assay

Complex IV (ferrocytochrome c:oxygen oxidoreductase, EC 1.9.3.1) enzymatic activity was measured in isolated mitochondria (10 µg) as described previously^59^, using a Cary 300 Bio single beam spectrophotometer (Varian, USA) at 30 °C. Complex IV activity (nmol/min/mg protein) was normalized to citrate synthase (EC 4.1.3.7) activity to correct for variations in total mitochondrial density^60, 61^. Three independent mitochondrial isolations were performed for each cell line and enzyme activity measurements were carried out in duplicate.

### RNA extraction

Total RNA was purified using a miRNeasy RNA extraction kit (Qiagen, Cat. #217004) with an on-column DNase I (Qiagen) treatment to eliminate contaminating genomic DNA.

### qRT-PCR analysis of mitochondrial transcripts

Complementary DNA (cDNA), synthesized using the QuantiTect Reverse Transcription Kit (Qiagen, Cat. #205311), was used as a template for subsequent quantitative real time reverse transcription PCR (qRT-PCR). qRT-PCR was performed using a StepOnePlus Real-Time PCR System with Fast Advanced master mix and TaqMan gene expression assays (Supplementary Table 2, Life Technologies) as described before^62^. Relative quantitation (fold RNA change) was obtained by applying the comparative Ct method^63^ whereby the mRNA expression of each mitochondrial transcript was normalized against the level of reference genes ACTB or HPRT1.

### Quantification of mtDNA copy number by quantitative PCR (qPCR)

Total DNA was purified using a QIAamp DNA Mini kit (Qiagen, Cat. #51304), with an on-column RNase A treatment to eliminate residual RNA. mtDNA copy number was measured by qPCR using *ACTB* as a marker of diploid genome content and mtDNA-encoded NADH dehydrogenase 2 (*MT-ND2*) as a marker of for the mitochondrial genome, in a StepOnePlus Real-Time PCR System (Life Technologies). Detailed information on preparation of *ACTB* and *MT-ND2* standards is described in Supplementary Information. TaqMan expression assays (Life Technologies) for VIC dye-labelled, primer-limited MT-ND2 (Hs02596874) and FAM dye-labelled ACTB (Hs03023880) were used in a single-tube, duplex qPCR reaction. Probes were selected and tested for non-specific amplification and each sample was run in triplicate in separate reactions. The number of mtDNA copies per cell was quantified using the following formula:

$${\rm{mtDNA}}\,\mathrm{copies}/\mathrm{cell}=({\rm{number}}\,{\rm{of}}\,{\rm{copies}}\,\mathrm{MT}-\mathrm{ND2}\,{\rm{gene}})/(\mathrm{number}\,{\rm{of}}\,{\rm{copies}}\,{\rm{ACTB}}\,\mathrm{gene}/2){\rm{.}}$$

### Statistical analysis

Statistical analysis was performed with GraphPad Prism software (GraphPad Software Inc.). One-way ANOVA or paired, two-tailed Student’s *t*-test were performed for comparison between experimental groups. Data in graphs are shown as mean ± standard deviation (SD). A *P-value < 0.05 was considered as statistically significant.

## Electronic supplementary material

Supplementary Information
