# Supplementary material for: Amyloid precursor protein drives down-regulation of mitochondrial oxidative phosphorylation independent of amyloid beta
Source: Sci Rep. 2017 Aug 29;7:9835. doi: 10.1038/s41598-017-10233-0 (PMC5574989; doi:10.1038/s41598-017-10233-0)
Supplement: Supplementary file 1 — Supplementary Information [file 41598_2017_10233_MOESM1_ESM.pdf]

Supplementary Information

Amyloid precursor protein drives down-regulation of mitochondrial oxidative phosphorylation independent of amyloid beta

M. Isabel G. Lopez Sanchez [#1,2], Hayley S. Waugh [#1,2], Andrew Tsatsanis[3], Bruce X. Wong[3,4], Jonathan G. Crowston [1,2] James A. Duce [3,4] and Ian A. Trounce [1,2,*]

[1] Centre for Eye Research Australia, 75 Commercial Road, Melbourne, 3004 Victoria, Australia.

[2] Ophthalmology, University of Melbourne, Department of Surgery.

[3] School of Biomedical Sciences, Faculty of Biological Sciences, University of Leeds, Leeds, West Yorkshire LS2 9JT, United Kingdom.

[4] Oxidation Biology Unit, The Florey Institute of Neuroscience and Mental Health, University of Melbourne, 30 Royal Parade, Parkville, 3052 Victoria, Australia.

[#] These authors contributed equally to this work.

[*] Corresponding author: Ian A. Trounce, Centre for Eye Research Australia, 75 Commercial Road, Melbourne, 3004 Victoria, Australia. E-mail: i.trounce@unimelb.edu.au.

Supplementary Methods

Sample preparation and SDS-PAGE

Total cellular protein was extracted by homogenization in RIPA buffer (50 mM Tris-HCl pH 7.4, 150 mM NaCl, 1 mM EDTA, 1% Tergitol and 0.1% SDS) supplemented with 10 µg/ml aprotinin, 2 mM Na_3VO_4 and 1 mM phenylmethylsulfonyl fluoride. Samples were incubated on ice for 30 min prior to centrifugation (18,000 *g*, 20 min, 4°C) and the supernatant retained. Cell lysates or mitochondrial preparations were heated at 95°C for 5 min (or 37°C for OXPHOS antibodies) and separated on 10% or 12% SDS-PAGE gels. Proteins transferred to nitrocellulose or PVDF membranes (Amersham, GE Healthcare) were blocked in 5% (w/v) skim milk in PBS-T (1X PBS, 0.05% Tween) for 1 h before incubation overnight at 4°C with primary antibodies.

Standard preparation for mtDNA copy number analysis

ACTB and MT-ND2 standards were produced by PCR using 100 ng DNA template, 1X PCR buffer, 100 µM dNTPs, 1.5 mM $MgCl_2$ (all from Invitrogen), 0.5 µM of each of the forward and reverse primers (ACTB forward 5'-GCAGAAGGAGATCACTGC-3' and reverse 5'-TAAAGCCATGCCAATCTC-3'; MT-ND2 forward 5'-ATTAATCCCCTGGCCCAA-3' and reverse 5'-GAAGGATTATGGATGCGGTT-3'), 2 U Taq Polymerase (Invitrogen) and ultrapure H_20 (Sigma-Aldrich), followed by purification of the PCR products using a GenElute PCR Clean-Up Kit (Sigma-Aldrich, Cat. # NA-1020), and sequenced to confirm the specific amplification of the MT-ND2 and ACTB targets. Standards were prepared by 10-fold serial dilution (1 x 10^{-2} ng/µl to 1 x 10^{-7} ng/µl) of the target-specific purified PCR product. MT-ND2 and ACTB probes were run individually and in duplex reactions using 10-fold dilution series of a control DNA sample to confirm reproducibility and that amplification efficiency was the same in single or duplex reactions.

Supplementary Tables and Figures

Supplementary Table 1. Antibodies used in this study

Antibody	Supplier	Catalogue #	Dilution used
Anti-Alzheimer precursor protein A4, N-term specific 22C11	Millipore	MAB348	1:2000
Anti-amyloid β antibody clone WO-2	Millipore	MABN10	1:2000 or 1:200
Anti-β-Amyloid, 1-16 antibody	BioLegend	803001	N/A
Anti-actin clone AC-40	Sigma-Aldrich	A3853	1:5000
Anti-VDAC1/Porin	Abcam	ab14734	1:5000
MitoProfile Total OXPHOS cocktail	MitoSciences	MS604	1:2000
Anti-SDHA	Abcam	ab14715	1:5000
Anti-COX2	Abcam	ab110258	1:5000
Anti-COX5A	Abcam	ab110262	1:5000
Anti-mouse horseradish peroxidase-conjugated secondary antibody	Amersham GE Healthcare	NA931V	1:10,000

Supplementary Table 2. Gene expression assays used in this study

Gene target	Assay catalogue #
MT-ND1	Hs02596873
MT-ND4	Hs02596876
MT-ND6	Hs02596879
MT-CO1	Hs02596864
MT-CO3	AIY9ZY3
MT-ATP6	Hs02596862
MT-CYB	Hs02596867
NDUFS3	Hs01549083
COX4I1	Hs00971639
ACTB	Hs03023880
HPRT1	Hs02800695

Supplementary Table 3. Respiration in control cells upon bIV treatment

	**Control untreated**	**Control + bIV**
Leak	9.32 ± 0.2	8.51 ± 1.7
CI	11.12 ± 0.5	8.40 ± 2.2
CI+II	13.88 ± 1.1	10.34 ± 2.2 *
Max	10.89 ± 0.8	9.12 ± 1.5

Convergent complex I+II respiration is decreased in control cells incubated with 1 µM β-secretase inhibitor IV (bIV) for 24 h relative to untreated control. Data is presented as mean ± SD (n = 3); * $P < 0.05$ by paired, two-tailed Student's *t*-test.

Supplementary Figure 1

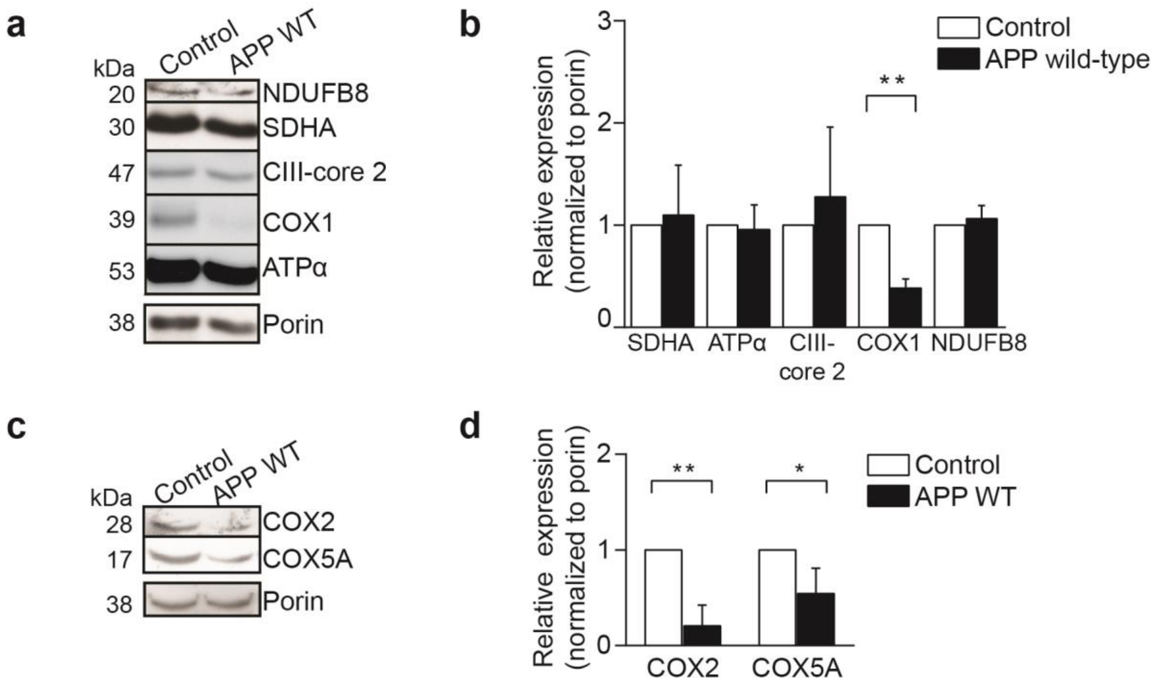

OXPHOS complex IV protein levels are decreased in mitochondria from APP wild-type cells

a) Representative immunoblot of OXPHOS protein levels in mitochondrial preparations from cells expressing APP wild-type (APP WT) compared to control. Porin was used as a loading control. **b)** Densitometric analysis of immunoblot images showing a significant decrease in the expression of mtDNA-encoded COX1. **c)** Representative immunoblot and **d)** Densitometric measurements show a significant decrease in the expression of additional complex IV protein subunits; mtDNA-encoded COX2 and nuclear DNA-encoded COX5A, in mitochondrial preparations from APP wild-type (APP WT) cells compared to control. Porin was used as a loading control. All data is presented as mean ± SD (n = 3); * $P < 0.05$ and ** $P < 0.01$ by paired, two-tailed Student's *t*-test.
